# Acorns containing deeper plumule survive better: how white oaks counter embryo excision by rodents

**DOI:** 10.1002/ece3.898

**Published:** 2013-12-11

**Authors:** Mingming Zhang, Zhong Dong, Xianfeng Yi, Andrew W Bartlow

**Affiliations:** 1 College of Agriculture, Henan University of Science and TechnologyLuoyang, 471003, China; 2 College of Food & Bioengineering, Henan University of Science and TechnologyLuoyang, 471003, China; 3 College of Life Sciences, Jiangxi Normal UniversityNanchang, 330022, China; 4 Department of Biology, University of UtahSalt Lake City, UT, 84112, USA

**Keywords:** Acorn germination, coevolution, embryo depth, embryo excision, white oak.

## Abstract

Several squirrel species excise the embryo of acorns of most white oak species to arrest germination for long-term storage. However, it is not clear how these acorns counter embryo excision and survive in the arms race of coevolution. In this study, we simulated the embryo excision behavior of squirrels by removing 4 mm of cotyledon from the apical end of white oak acorns differing in embryo depths to investigate the effects of embryo excision on acorn germination and seedling performance of white oak species. The embryo depth in the cotyledons was significantly different among white oak acorns, with *Quercus mongolica* containing the embryo most deeply in the acorns. We found that artificial embryo excision significantly decreased acorn germination rates of *Quercus variabilis*, *Quercus acutissima*, *Quercus aliena*, *Quercus aliena var. acutiserrata*, *Quercus serrata. var. brevipetiolata* but not *Q. mongolica*. Artificial embryo excision exerted significant negative impacts on seedling performance of all oak species except *Quercus aliena*. Our study demonstrates the role of embryo depth of acorns in countering embryo excision by squirrels and may explain the fact that squirrels do not perform embryo excision in acorns of *Q. mongolica* with deeper embryos. This apparent adaptation of acorns sheds light on the coevolutionary dynamics between oaks and their seed predators.

## Introduction

Acorns of most early germinating white oak species (subgenus *Quercus*) exhibit no dormancy and germinate immediately at seed fall, rapidly moving nutritional stores into robust taproots in autumn (Barnett 1977; Fox 1982; Hadj-Chikh et al. 1996; Steele et al. 2001a, 2006). This strategy is widely regarded as an adaptation to seed predation by small rodents (Fox 1982; Jansen et al. 2006; Xiao et al. 2010, 2013a; Sung et al. 2010; Cao et al. 2011). However, several rodent species selectively store red oak acorns over those of white oaks (Smallwood et al. 2001; Steele et al. 2001a), specifically in response to differences in perishability due to different germination schedules (Hadj-Chikh et al. 1996; Steele et al. 2001b; Chang et al. 2009). To maximize rewards from caches, at least three species of squirrels (*Callosciurus erythraeus*, *Dremomys rufigenis,* and *Sciurotamias davidianus*) in Asia (Xiao et al. 2009, 2010; Xiao and Zhang 2012) and three (*Sciurus carolinensis*, *S. aureogaster*, *S. niger*) in North America (Fox 1982; Steele et al. 2001b; Moore 2005; Steele 2008) are known to excise the plumule (hereafter termed embryo) of white oak acorns (*Quercus variabilis*, *Quercus aliena var. acutiserrata*, *Quercus serrata. var. brevipetiolata*, *Quercus alba*, and *Quercus montana*) before storing them. Recently, Xiao et al. (2013a,b) further found that embryo excision behavior by squirrels was much more common in large acorns and in seed masting years than in small acorns and seed poor years. Previous studies have emphasized the negative influences of embryo excision on acorn germination; however, it is not clear how these acorns counter embryo excision behavior by squirrels (McEuen and Steele 2005; Xiao et al. 2010). Although observations suggest a strong evolutionary relationship between rodents and oaks, we still have a poor understanding of the adaptive traits of white oak acorns that may be responsible for resisting embryo excision in this interaction.

**Figure 1:**
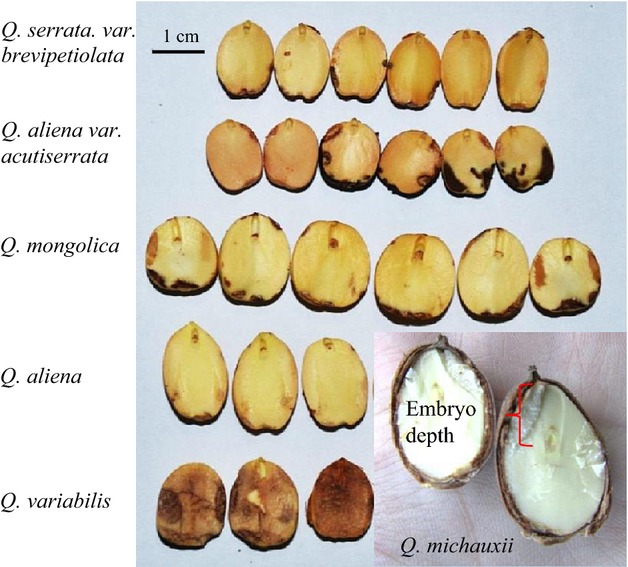
Longitudinal sections of acorns of several white oaks native to China (excluding *Q. michauxii*). Shown are the plumule position and depth.

Coevolution occurs when two or more species influence each other's evolution and is most often invoked to explain coadaptations between species (Kiester et al. 1984; Thompson 2005). White oak acorn embryo depth varies significantly at intra- and interspecific levels, ranging from 0.3 cm to 1.30 cm from the apical end of the acorns (Fig. 1). However, we still have little knowledge of how acorns of white oaks cope with embryo excision by rodents (Yi et al. 2012, 2013a; Xiao et al. 2010, 2013a,b). Embryo excision behavior by squirrels is often found in acorns of white oak species with shallow embryos (Steele et al. 2001b; Xiao et al. 2009, 2010; Xiao and Zhang 2012); however, no evidence of excision is found in those containing deep embryos (e.g., *Q. mongolica* and *Q. michauxii*). Here, we hypothesize that if the embryo position in the cotyledons plays an important role in countering embryo excision, acorns with deep embryos will have a greater ability to cope with embryo excision and be less likely to be excised by squirrels. In this study, we explored the effects of artificial embryo excision on acorn germination and seedling performance of the Asian white oak species *Quercus variabilis*, *Q. acutissima*, *Q. mongolica*, *Q. aliena*, *Q. aliena var. acutiserrata*, and *Q. serrata. var. brevipetiolata* in an attempt to determine the role of embryo position in acorns in protecting embryos against excision by tree squirrels and in supporting acorn germination and seedling establishment.

## Methods

### Collection of acorns and measurement of embryo position

We collected acorns of *Q. variabilis*, *Q. aliena*, and *Q. serrata. var. brevipetiolata* in Tianchi Mountain National Forest Park, Henan province, China (33°45′–33°85′N, 111°75′–112°45′E). *Quercus acutissima* acorns were collected in Zijin Mountain, Nanjing, China (32°04′N, 118°49′E), 700 km southeast of Tianchi Mountain National Forest Park. Acorns of *Q. aliena var. acutiserrata* were collected in Qinling Mountain, Shaanxi province, China (35°0′N, 105°30′E), 400 km southwest of Tianchi Mountain National Forest Park. Embryo excision behavior of squirrels has been reported or observed in these areas (Xiao et al. 2009, 2010; Dong 2013). Acorns of the unique oak species *Q. mongolica* were collected in Xiaoxing'anling Mountain, Heilongjiang province, China (46°50′–46°59′N, 128°57′–129°17′E), 1900 km northeast of Tianchi Mountain National Forest Park. Acorns of these oak species were randomly collected from several trees in 2012 to make a composite sample. Twenty acorns of each oak species were randomly selected to measure the seed dimensions (length and width) and embryo depth. Embryo depth was defined as the distance between the apical end of the cotyledons and the base of the plumule (Fig. 1) and measured using a vernier caliper to the nearest 0.01 cm. Fifteen acorns of each oak species were randomly selected and dried at 70°C for 48 h for dry mass measurements.

### Effects of artificial embryo excision on acorn germination and seedling establishment

One hundred acorns of each oak species were randomly selected and sorted into two groups each with 50 acorns. One group was treated as control, while the embryos of 50 acorns of the other group were artificially excised. Simulating the behavior of squirrels, we cut off 4 mm of the cotyledon from the apical end of each acorn according to the incisor length (5–6 mm, measured from the gingival margin to the coronal tip) of red squirrels (Sainsbury et al. 2004) and the depth of the holes (0.48 ± 0.12 cm, *n* = 10) remaining in *Q. variabilis* acorns excised by squirrels. We then sowed these acorns into plastic trays with a 5 × 10 grid containing organic composite soil. Plastic trays were kept in the laboratory at room temperature (20–25°C) and subject to visible light 800 *μ*mol m^−2^ s^−1^ radiation under a 14-h photoperiod. Plant containers were regularly watered to keep moist. An acorn was considered germinated when the epicotyl emerged, while time to germination was defined as the time when the first epicotyl emerged in each acorn group since sowing. Germination rates were recorded at irregular intervals after sowing. Dry masses of seedling parts, dried at 70 °C for 48 h, were measured 132 days after sowing.

### Seed dispersal of *Quercus mongolica* acorns by red squirrels

*Quercus mongolica* is the unique oak species in Xiaoxing'anling Mountain areas in northeastern China, and red squirrels *Sciurus vulgaris* are considered to be the main dispersers of its acorns (Hao and Wu 2012). To test whether red squirrels perform embryo excision behavior on *Q. mongolica* acorns with deep embryo, we randomly selected 1000 acorns of *Q. mongolica* and labeled them with plastic tags and thin steel threads according to Xiao and Zhang (2012). Tagging shows no significant influence on seed caching and embryo excision behavior of squirrels (Xiao et al. 2009, 2010; Xiao and Zhang 2012). This method allows us to easily locate the scatter-hoarded acorns after dispersal by squirrels. In October 2012, we established 10 arenas (0.8 m high, 100 m apart) and provided 100 tagged acorns in each arena in a mature secondary forest in Xiaoxing'anling Mountain areas. Each arena was covered by steel net mesh (1 cm × 1 cm) to prevent avian removal of acorns. We only wanted to document red squirrel acorn removal and dispersal; however, Eurasian jays *Garrulus glandarius* had limited access to these acorns. After acorn release, seed removal was checked every day and seed fates were placed into one of six categories according to previous observations (Yang and Yi 2012): (1) remaining in situ (R), (2) eaten in situ (EIS), (3) eaten after removal (EAR), (4) intact after removal (on ground surface) (IAR), (5) cached after removal (in the soil or litter) (CAR), and (6) missing. For the cached acorns, we carefully dug them out to check whether their embryos were excised by squirrels.

### Data analysis

Statistical Package for the Social Sciences (SPSS 16.0; IBM SPSS, Inc., Chicago, IL) and the statistical program R (R Core Team 2012) were used for the data analyses. MANOVA was used to detect differences in acorn dimensions, dry masses, and embryo depths among the white oak species. To determine whether the dependent variable was normally distributed for each combination of the levels of the two independent variables, Levene's Test was used. Differences in final germination status and time to germination of acorns were detected using chi-squared tests and sequential Bonferroni corrections to avoid significance errors. P values are reported and were compared to sequential Bonferroni-corrected significance levels (*N* = 6) to minimize type I error. Differences in dry masses of seedling parts originated from the intact acorns, and those with embryos excised were tested using the nonparametric Mann–Whitney *U*-test.

## Results

### Acorn traits

Our results showed significant differences in seed dimensions of the six white oak species in terms of acorn length (*F*_5,114_ = 19.051, df = 5, *P* < 0.001) and width (*F*_5,114_ = 58.981, *P* < 0.001) (Table 1). Consistent with the acorn dimensions, the dry masses of acorns were significantly different among the six white oaks species (*F*_5,84_ = 30.927, *P* < 0.001) (Table 1). *Quercus acutissima* and *Q. variabilis* produced larger acorns, *Q. mongolica* and *Q. aliena* were oak species bearing medium-sized acorns, and *Q. aliena var. acutiserrata* and *Q. serrata. var. brevipetiolata* were characterized by small-sized acorns. The embryos of *Q. mongolica* acorns were embedded deepest in the cotyledons compared with other oak species, while *Q. aliena var. acutiserrata* and *Q. serrata. var. brevipetiolata* were characterized by cotyledons containing the shallowest embryos (*F*_5,114_ = 37.648, *P* < 0.001) (Table 1).

**Table 1:** Seed dimensions and embryo depths in acorns of several white oak species in China. MANOVA was used to detect differences in acorn traits

Oak species	Length (cm) (*n* = 20)	Embryo depth (cm) (*n* = 20)	Width (cm) (*n* = 20)	Dry mass (g) (*n* = 15)
*Quercus mongolica*	1.91 ± 0.04^bc^	0.76 ± 0.04^a^	1.49 ± 0.04^ac^	1.90 ± 0.57^c^
*Quercus variabilis*	2.01 ± 0.06^ab^	0.63 ± 0.03^a^	1.63 ± 0.04^a^	3.02 ± 1.00^b^
*Quercus aliena*	2.20 ± 0.07^a^	0.53 ± 0.02^b^	1.43 ± 0.03^bc^	1.72 ± 0.83^c^
*Quercus acutissima*	1.92 ± 0.04^bc^	0.44 ± 0.01^c^	1.60 ± 0.04^a^	3.56 ± 0.91^a^
*Quercus aliena* var*. acutiserrata*	1.54 ± 0.04^d^	0.32 ± 0.01^d^	1.08 ± 0.02^d^	1.17 ± 0.38^d^
*Quercus serrate* var*. brevipetiolata*	1.70 ± 0.05^d^	0.30 ± 0.01^d^	1.02 ± 0.02^d^	1.03 ± 0.18^d^

Different letters in the same column indicate significance at *α* = 0.05 level.

### Acorn germination and seedling performance

**Figure 2:**
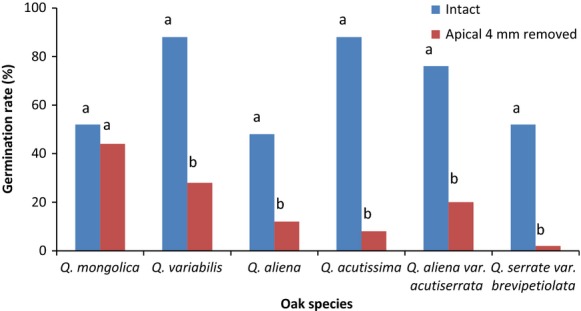
Percentage of Chinese oak acorns germinated in response to artificial embryo excision. Differences were detected using chi-squared tests, and sequential Bonferroni corrections were used to adjust the significance levels. Different letters on bars of the same oak species indicate significance.

**Figure 3:**
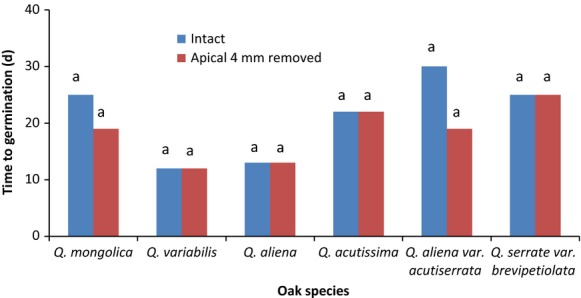
Time to germination (d) of Chinese oak acorns in response to artificial embryo excision. Differences were detected using chi-squared tests and sequential Bonferroni corrections were used to adjust the significance levels. Different letters on bars of the same oak species indicate significance.

Artificial embryo excision significantly decreased the germination of acorns of *Q. variabilis*, *Q. acutissima*, *Q. aliena*, *Q. aliena var. acutiserrata*, and *Q. serrata. var. brevipetiolata* (*χ*^2^ = 34.52, df = 1, *P* < 0.0001; *χ*^2^ = 60.94, df = 1, *P* < 0.0001; *χ*^2^ = 13.76, df = 1, *P* < 0.001; *χ*^2^ = 29.21, df = 1, *P* < 0.0001; *χ*^2^ = 46.16, df = 3, *P* < 0.0001) (Fig. 2). These P values were less than the sequential Bonferroni-adjusted significance levels; and therefore, inferences made from the data did not change. However, acorn germination of *Q. mongolica* was not significantly influenced by artificial embryo excision (*χ*^2^ = 0.3606, df = 1, *P* = 0.548) (Fig. 2). We also found a close correlation between embryo depths and germination rates of excised acorns among the six white oak species (*r* = 0.839, *n* = 6, *P* = 0.042). In addition, artificial embryo excision did not alter the time to germination of acorns of *Q. mongolica*, *Q. variabilis*, *Q. acutissima*, *Q. aliena*, *Q. aliena var. acutiserrata*, and *Q. serrata. var. brevipetiolata* (*χ*^2^ = 0.818, df = 1, *P* = 0.366; *χ*^2^ = 0.000, df = 1, *P* = 1.00; *χ*^2^ = 0.000, df = 1, *P* = 1.00; *χ*^2^ = 0.000, df = 1, *P* = 1.00; *χ*^2^ = 2.469, df = 1, *P* = 0.116; *χ*^2^ = 0.000, df = 1, *P* = 1.00) (Fig. 3). The P values of all the six species were greater than the sequential Bonferroni-adjusted significance levels. However, there were negative impacts on seedling performance in terms of seedling height, leaf number, and above- and belowground dry mass (Table 2).

**Table 2:** Effects of artificial embryo excision on seedling performance of white oaks in China. Mann–Whitney *U*-tests were used to detect differences in seedling performance

Acorn Type	Oak species	Seedling height (cm)	Leaf number (*N*)	Belowground dry mass (g)	Aboveground dry mass (g)
Intact acorns	*Quercus mongolica*	8.58 ± 1.76^a^	3.17 ± 0.65^a^	0.33 ± 0.08^a^	0.16 ± 0.09^a^
	*Quercus variabilis*	24.27 ± 1.07^a^	5.30 ± 0.28^a^	0.32 ± 0.02^a^	0.43 ± 0.03^a^
	*Quercus aliena*	9.96 ± 0.94^a^	2.92 ± 0.50^a^	0.19 ± 0.02^a^	0.22 ± 0.04^a^
	*Quercus acutissima*	23.43 ± 1.05^a^	4.40 ± 0.61^a^	0.27 ± 0.03^a^	0.57 ± 0.04^a^
	*Quercus aliena* var*. acutiserrata*	10.76 ± 0.65^a^	3.72 ± 0.34^a^	0.14 ± 0.02^a^	0.20 ± 0.02^a^
	*Quercus serrate* var*. brevipetiolata*	3.50 ± 0.73^a^	1.14 ± 0.51^a^	0.19 ± 0.02^a^	0.09 ± 0.02^a^
Apical 4 mm removed acorns	*Q. mongolica*	5.34 ± 0.91^b^	3.20 ± 0.83^a^	0.13 ± 0.05^b^	0.09 ± 0.03^b^
	*Q. variabilis*	26.31 ± 3.82^a^	5.57 ± 0.97^a^	0.17 ± 0.04^b^	0.25 ± 0.04^b^
	*Q. aliena*	12.17 ± 3.67^a^	1.67 ± 0.88^a^	0.13 ± 0.08^a^	0.15 ± 0.09^a^
	*Q. acutissima*	11.25 ± 9.25^b^	4.50 ± 4.5^a^	0.19 ± 0.11^a^	0.35 ± 0.28^a^
	*Q. aliena* var*. acutiserrata*	10.87 ± 1.53^a^	2.43 ± 0.48^b^	0.11 ± 0.03^a^	0.12 ± 0.03^b^
	*Q. serrate* var*. brevipetiolata*	1.12 ± 0.23^b^	0.00 ± 0.00^b^	0.01 ± 0.01^b^	0.01 ± 0.02^b^

Different letters in the same column indicate significance at *α* = 0.05 level between the same oak species.

### Acorn dispersal by red squirrels

**Figure 4:**
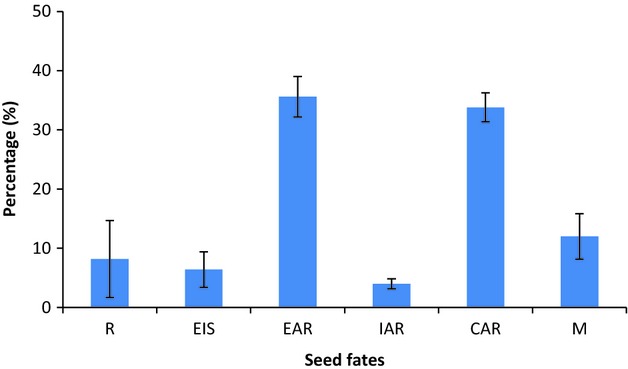
The mean percentage (± SE) of *Quercus mongolica* acorns of each fate after dispersal by red squirrels in the field in northeastern China. The six possible fates were remaining in situ (R), eaten in situ (EIS), eaten after removal (EAR), intact after removal (on ground surface) (IAR), cached after removal (in the soil or litter) (CAR), and missing (M).

Among the 1000 *Q. mongolica* acorns we released in the arenas, 92.8% were removed or consumed, with 33.8% being scatter hoarded. Although we recovered all 338 acorns from caches, none of them had their embryos excised by red squirrels (Fig. 4).

## Discussion

Our results demonstrate that artificial embryo excision to 4 mm reduces acorn germination rates of *Q. variabilis*, *Q. acutissima*, *Q. aliena*, *Q. aliena var. acutiserrata*, and *Q. serrata. var. brevipetiolata*, verifying the negative effects of embryo excision by squirrels on acorn germination of white oaks (Steele et al. 2001b; Xiao et al. 2009, 2010). Embryo excision arrests acorn germination and facilitates long-term acorn storage and ensures more food rewards from caches by squirrels. Our results on *Q. variabilis* acorns are very similar to those of Xiao and Zhang (2012) that showed embryo excision by three squirrel species (*Callosciurus erythraeus*, *Dremomys rufigenis,* and *Sciurotamias davidianus*) significantly decreases acorn germination rates of *Q. variabilis*. This indicates that our acorn manipulations in this study successfully simulated embryo excision behavior of squirrels. These results imply that the embryos in acorns of *Q. variabilis*, *Q. acutissima*, *Q. aliena*, *Q. aliena var. acutiserrata*, and *Q. serrata. var. brevipetiolata* are not deep enough to cope with simulated embryo excision. However, acorn germination rates of *Q. mongolica* were not affected by artificial embryo excision to 4 mm in our study, which can be attributed to the typical embryo position in acorns of *Q. mongolica*. Acorns with deeper embryos are expected to have a higher probability of escaping embryo excision by squirrels at intra- and interspecific levels. While partial damage influences acorn germination (Hou et al. 2010; Perea et al. 2011; Mancilla-Leytón et al. 2012, 2013; Yang and Yi 2012), we did not observe a similar effect on time to germination of the acorns of the six oak species. This contradiction cannot be explained by faster water uptake in partially damaged acorns (Kikuzawa and Koyama 1999) or by faster export from edible tissues and greater partitioning into tissues inaccessible to foraging herbivores (Orians et al. 2011) and remains unexplained. Artificial embryo excision shows significant impacts on oak seedling performance, which can be explained by cotyledon losses (Yang and Yi 2012; Mancilla-Leytón et al. 2012), especially the essential part at the apical end of acorns (Hou et al. 2010).

In the coevolutionary history between plants and seed eating animals, attack and counterattack impose strong selection on seeds and their predators, resulting in the development of reciprocal evolutionary dynamics (Kanzaki et al. 2012). In the oak dispersal system, acorns of many white oaks (e.g., subgenera *Quercus* and *Cerris*), in contrast to those of red oaks (subgenus *Lobatae*), exhibit no dormancy and germinate immediately at or even before seed fall, rapidly producing robust taproots (Barnett 1977; Steele et al. 2001a, 2006). This strategy has been regarded as an adaptive strategy of white oak acorns to survive seed predation by small rodents (Fox 1982; Xiao et al. 2010; Yi et al. 2012, 2013a). While prey species evolve successful defenses, predator species also evolve techniques for better foraging (Xiao et al. 2007; Tamura 2011; Toju et al. 2011; Yi et al. 2013b). Several squirrel species appear to retard or completely arrest germination of acorns by removing the entire embryo (Fox 1982; Steele et al. 2001b; Xiao et al. 2009, 2010; Xiao and Zhang 2012), allowing squirrels to use nondormant acorns as a long-term food supply. However, results in this study clearly show that artificial embryo excision exerts no significant effects on acorn germination of *Q. mongolica*. Moreover, the deep embryos in *Q. mongolica* acorns seem to prevent embryo excision behavior by red squirrels. Although several oak species with shallow embryos have been reported to be embryo-excised by various tree squirrels (Fox 1982; Steele et al. 2001b; Xiao et al. 2009), *Q. mongolica* acorns with deep embryo are not found to be embryo-excised in the field. Despite the delayed germination schedule, acorns of *Castanea henryi* and *Q. rubra* also suffered from embryo excision by squirrels (Steele et al. 2001b; Xiao et al. 2009), possibly due to the shallow embryos in their cotyledons. These observations suggest that *Q. mongolica* has evolved a specialized defense system (deep embryo) in northeastern China in response to embryo excision behavior by red squirrels, which has been subsequently lost. Differing from other oak acorns with shallow embryos, germinating acorns of *Q. mongolica* with deep embryos are characterized by elongation and swelling of the hypocotyls rather than cotyledonary petioles (0.5–1 cm) (Yi et al. 2012). This hypocotyl results in two split cotyledons attached to the base of seedlings. Deep embryos in acorns are expected to facilitate *Q. mongolica* seedlings in escaping rodent predation as it is easy for rodents to detach the cotyledons from anchored taproots of young seedlings. Moreover, a previous study showed that no acorns were embryo-excised by red squirrels when provided with acorns of alien oak species *Q. variabilis*, *Q. aliena*, and *Q. serrata. var. brevipetiolata* containing shallow embryos (Lei et al. 2012; X. F. Yi, unpubl. data). Our results seem to challenge the prediction of Xiao et al. (2009) that embryo excision would be a convergent behavior of tree squirrels. The absence of embryo excision behavior of red squirrels implies the advantage of *Q. mongolica* acorns during the long-term coevolutionary history between oaks and squirrels. Squirrel species in other areas may have overcome the specialized defense system of early acorn germination of white oaks with shallow embryos (Xiao et al. 2009, 2010).

To our knowledge, this is the first study to verify the effect of embryo depth in acorns of white oak species on acorn germination and embryo excision behavior of squirrels. This represents an evolutionary tactic of white oak acorns to cope with predation by small rodents. Our results show that artificial embryo excision exerts different impacts on acorn germination of white oak species, suggesting that embryo depth plays a significant role in countering embryo excision by their mutualistic partner and can influence embryo excision behavior of squirrels. However, acorns of the closest relative of *Q. mongolica*, *Q. liaotungensis*, contain shallow embryos and are not embryo-excised by squirrels (Zhang et al. 2008), suggesting that the embryo position in acorns is not the only determinant of embryo excision. Although it is not clear why only acorns of *Q. mongolica* contain deep embryo, a possible explanation is that deep embryos (plumules) in acorns are expected to be well protected under extremely low temperatures (−40°C) in winter in northeastern China (Yang and Yi 2012) where *Q. mongolica*, rather than other oak species, are widely distributed. It should be noted that the existing embryo excision behavior by squirrels is often found in the forests where red oaks (subgenus *Erythrobalanus*) or ginggang oaks (subgenus *Cyclobalanopsis*) co-occur with white oaks (Fox 1982; Steele et al. 2001b; Moore 2005; Steele 2008; Xiao et al. 2009, 2010, 2013a,b), and we still lack evidence of this behavior in forests where only white oaks occur (Zhang et al. 2008; Yang and Yi 2012; and this study). Moreover, squirrels excise the embryo of white oak acorns in old forests where white oaks have long been extirpated (Xiao and Zhang 2012). It is plausible that past or current coexisting oaks bearing dormant and nondormant acorns is a prerequisite for the evolution of this type of behavior of squirrels. We propose that long-term experience with and/or recognition of different germination schedules in white and red oaks are expected to select for embryo excision behavior of squirrels, which needs further investigation.
